# Morphology and immunophenotype of canine cutaneous histiocytic tumours with particular emphasis on diagnostic application

**DOI:** 10.1007/s11259-014-9622-1

**Published:** 2014-11-25

**Authors:** Katarzyna Paździor-Czapula, Tadeusz Rotkiewicz, Iwona Otrocka-Domagała, Michał Gesek, Anna Śmiech

**Affiliations:** 1 Department of Pathological Anatomy, Faculty of Veterinary Medicine, University of Warmia and Mazury in Olsztyn, Oczapowskiego 13, 10-719 Olsztyn, Poland; 2 Department of Pathological Anatomy, Faculty of Veterinary Medicine, University of Life Sciences in Lublin, Głęboka 30, 20-612 Lublin, Poland

**Keywords:** Canine cutaneous histiocytoma, Histiocytic sarcoma, MHCII, CD18, E-cadherin, Regression

## Abstract

This study evaluated the morphology and immunohistochemistry of 85 canine cutaneous histiocytic tumours. The tumours were classified morphologically as either canine cutaneous histiocytomas (71 tumours) or canine cutaneous histiocytic sarcomas (14 tumours). The immunohistochemical analysis was conducted on paraffin sections using an antibody panel (against MHCII, CD18, CD79αcy, CD3 and E-cadherin). Histochemical staining with toluidine blue and Gomori silver impregnation was also performed. A follow-up was conducted via surveys. The histiocytic origin of the tumour cells was confirmed in 65 of the canine cutaneous histiocytomas and in 4 of the canine cutaneous histiocytic sarcomas. The tumours that had been misdiagnosed as canine cutaneous histiocytomas included plasmacytomas, epitheliotropic T-cell lymphomas and undetermined entities. The tumours misdiagnosed as canine cutaneous histiocytic sarcomas included plasmacytomas and non-epitheliotropic T-cell lymphomas, but the majority of them remained undetermined. The canine cutaneous histiocytomas showed MHCII, CD18 and E-cadherin expression, but in several of the tumours, the expression of CD18 or E-cadherin was confirmed in only a small percentage of the tumour cells. The regressing canine cutaneous histiocytomas showed increased T- and B-lymphocyte infiltration, a decreased mitotic index, transport of the MHCII molecules from the cytoplasm to the cell membrane and loss of E-cadherin expression in the tumour cells. The canine cutaneous histiocytic sarcomas showed both high morphological diversity and expression of MHCII and CD18. Two of the evaluated histiocytic sarcomas also showed expression of E-cadherin. In conclusion, immunohistochemistry, including analysis of MHCII, CD18 and the lymphocytic markers CD3 and CD79, should be performed for the diagnosis of canine cutaneous histiocytic tumours. The expression of E-cadherin in canine cutaneous histiocytic sarcomas suggests an origin of the tumour cells among Langerhans cells.

## Introduction

Canine cutaneous histiocytoma (CCH) is a common benign skin tumour of the dog, characterised by clonal proliferation of Langerhans cells and showing expression of CD1 and specific β2 integrins (Moore et al. 1996; Delcour et al. 2013). CCH may have a similar morphologic appearance as other round cell tumours; therefore, differentiation among CCH, plasmacytomas, mast cell tumours and cutaneous lymphomas based on histopathology alone is often challenging (Fernandez et al. 2005). In CCH, intra-epidermal nests of histiocytes are frequently noted, which resemble Pautrier’s aggregates characteristic of epitheliotropic lymphoma (Moore et al. 1996). Furthermore, the large number of lymphocytes in regressing CCH make differentiation between CCH and non-epitheliotropic lymphoma very difficult (Moore 2014). Therefore, the diagnosis of CCH should be based on the immunophenotype of the tumour cells. The most specific marker for Langerhans cells is the CD1a molecule, which is also expressed by the cortical thymocytes and dendritic cells of the thymic medulla. Unfortunately, analysis of this marker requires snap-frozen tissue samples and cannot be used in routine histopathology (Moore et al. 1996; Fernandez et al. 2005). The most reliable criteria for the immunohistochemical diagnosis of CCH when only paraffin sections are available are CD18 and MHCII positivity coupled with negative labelling for the lymphocytic markers CD3 and CD79α (Fernandez et al. 2005). Furthermore, the expression of E-cadherin is a valuable indicator of Langerhans cells’ differentiation in CCH, but the true incidence of E-cadherin expression in CCH is not known (Moore 2014).

The unique feature of CCH is its spontaneous regression, which occurs within 2–3 months (Schwens et al. 2011). The factors determining the onset of CCH regression are unknown (Moore et al. 1996). The spontaneous regression of the AK-5 histiocytoma in the rat is mediated by NK cells through antibody-dependent cellular cytotoxicity, and target cell death involves necrosis and apoptosis (Khar et al. 1997). The regression of CCH is associated with infiltration by CD8 (cytotoxic) lymphocytes and increased expression of IL-2, TNFα, IFNγ and iNOS mRNA (Kaim et al. 2006). CCH regression is correlated with lymphocytic infiltration, transport of MHCII molecules to the cell surface and loss of E-cadherin expression (Cockerell and Slauson 1979; Kipar et al. 1998; Pires et al. 2009). The regression process primarily involves T-lymphocytes, but a recent report also noted an increase in B-lymphocytes in regressing CCH (Pires et al. 2013b). However, the incidence of granulocytes in relation to the CCH regression was not evaluated.

Canine histiocytic sarcoma, which is derived from dendritic cells, occurs in a localised and disseminated form. Localised histiocytic sarcoma is typically situated subcutaneously on the limbs; other locations include the spleen, tongue, lung, brain stem, nasal cavity, vertebral bone and epidural space. Disseminated histiocytic sarcomas are most often found in the spleen, liver, lung, bone marrow and lymph nodes (Affolter and Moore 2002). Histiocytic sarcomas derived from splenic and bone marrow macrophages are characterised by both tumour cells with distinct haemophagocytic activity and an aggressive clinical course (Moore et al. 2006). Cutaneous histiocytic sarcomas most likely originate from the interstitial dermal dendritic cells and show strong pleomorphism. It is typical for large round cells, spindle cells and multinucleated giant cells to be present within one tumour (Affolter and Moore 2002; Constantino-Casas et al. 2011). The spindle-cell-predominant histiocytic sarcoma can mimic other pleomorphic sarcomas, whereas the round-cell-predominant histiocytic sarcoma should be differentiated from Langerhans cell histiocytosis, poorly granulated mast cell tumours and amelanotic melanomas (Lee Gross et al. 2005). Due to the broad range of morphologic features observed in histiocytic sarcomas, an immunophenotypic evaluation is necessary to confirm the diagnosis (Affolter and Moore 2002). The tumour cells of canine histiocytic sarcomas consistently express CD18, MHCII, CD45, CD1 and CD11c but are lacking in expression of E-cadherin (Lee Gross et al. 2005).

The first aim of the present study was to perform a detailed evaluation of the morphology of and MHCII, CD18 and E-cadherin immunoexpression in CCH and canine cutaneous histiocytic sarcoma. The second aim of the study was to evaluate changes in the immunophenotype of regressing CCH and to assess the contribution of particular inflammatory cell types to the spontaneous regression of CCH.

## Materials and methods

Cutaneous tumours were collected from 85 dogs by surgical excisional biopsies. All of the tissue samples were immediately fixed in 10 % buffered formalin, embedded in paraffin wax, cut into 3 μm sections and mounted on silanised glass. The sections were processed routinely and stained with Mayer’s haematoxylin and eosin (HE). The tumours included 71 CCHs and 14 cutaneous histiocytic sarcomas. The tumours were diagnosed on the basis of the histopathological examination according to widely used criteria (Lee Gross et al. 2005). The immunohistochemical examination of each tumour was performed using an antibody panel (against MHCII, CD18, CD79αcy, CD3, and E-cadherin) and a visualisation system based on the immunoperoxidase method, with 3,3-diaminobenzidine (DAB) as a substrate (Table 1). The specimens were counterstained with Mayer’s haematoxylin, dehydrated and mounted using Canadian balm. For the negative control, the primary antibody was either replaced with mouse IgG2a (Dako, Glostrup, Denmark) at the appropriate dilution (MHCII, CD18, CD79αcy, E-cadherin) or omitted (CD3). For the positive control, normal canine tonsil (MHCII, CD3, CD79αcy), canine cutaneous purulent inflammation (CD18) and normal canine skin (E-cadherin) sections were processed together with the evaluated sections. The presence of mast cells was confirmed by toluidine blue staining (Sigma-Aldrich, St. Louis, MO). Gomori’s silver impregnation method for the reticulum was used to detect reticulin fibres (staining kit, Bio-Optica, Milan, Italy).

**Table 1:** Primary antibodies, antigen retrieval and visualisation systems

Primary antibody	Clone	Dilution	Antigen retrieval	Visualisation system
HLA-DR α chain (MHCII)^a^	monoclonal mouse anti-human TAL.1B5	1:20	2x3 min.^b^ citrate buffer pH 6	EnVision System-HRP, Mouse (DAB)^a^
CD18^c^	monoclonal mouse anti-canine CA16.3C10	1:10	5 min. proteinase K^a^	EnVision System-HRP, Mouse (DAB)^a^
CD3^a^	polyclonal rabbit anti-human	1:50	2x3 min.^b^ Tris-EDTA buffer pH 9	ImmPRESS Universal Reagent Anti-Mouse/Rabbit Ig Peroxidase^d^
CD79αcy^a^	monoclonal mouse anti-human HM57	1:25	4x3 min.^b^ Tris-EDTA buffer pH 9	EnVision System-HRP, Mouse (DAB)^a^
E-cadherin^a^	monoclonal mouse anti-human NCH-38	1:50	2x3 min.^b^ Tris-EDTA buffer pH 9	EnVision System-HRP, Mouse (DAB)^a^

^a^Dako, Glostrup, Denmark

^b^Antigen retrieval was conducted in a microwave oven (650 W)

^c^PF. Moore, Davis, CA

^d^Vector Laboratories Inc., Burlingame, CA

Microscopic evaluation of the slides was conducted using the Pannoramic MIDI scanner (3DHISTECH, Budapest, Hungary) and Pannoramic Viewer software (3DHISTECH, Budapest, Hungary). The histiocytic origin of the tumour cells was confirmed by immunohistochemistry (MHCII+, CD18+, CD79αcy-, CD3-) according to Fernandez et al. (2005). A detailed morphological examination was performed using slides stained with HE. The morphologic analysis included assessment of the density of the cellular infiltration, the cellular and nuclear size and shape, epithelial changes (ulceration, parakeratosis, hydropic degeneration of keratinocytes, intra-epidermal neoplastic infiltration), other changes (oedema, necrosis, angiogenesis) and the mitotic index. The mitotic index was assessed by direct counting of the fraction of cell nuclei in the ‘M’ phase of the cell cycle (Bacchi and Gown 1993). The mitotic figures were evaluated on the whole slide, and the mitotic index was defined as the maximal number of mitotic figures per high-power field (HPF) (magnification 400x). The inflammatory infiltrate was evaluated on a scale of 1–4 points (1 = single cells; 2 = small foci of 3–9 cells; 3 = large foci of more than 9 cells; 4 = focal and diffuse) using slides stained with HE (neutrophils, eosinophils), toluidine blue (mast cells), CD3 immunohistochemical labelling (T-lymphocytes) and CD79αcy immunohistochemical labelling (B-lymphocytes, plasma cells). Additionally, for CCHs, the pattern of the lymphocytic infiltration was described as minimal, moderate, marked or massive, according to the classification of Cockerell and Slauson (1979). The expression of MHCII, CD18 and E-cadherin in the histiocytic cells was evaluated quantitatively on a scale of 1–4 points (1 = less than 20 % positive histiocytic cells; 2 = 20–50 % positive histiocytic cells; 3 = 51-80 % positive histiocytic cells; 4 = more than 80 % positive histiocytic cells). Additionally, the MHCII expression pattern was evaluated qualitatively on a scale of 1–4 points (1 = cytoplasmic expression; 2 = predominantly cytoplasmic expression; 3 = predominantly membranous expression; 4 = membranous expression). Microphotographs were prepared using a microscope equipped with a DP73 digital camera (Olympus, Tokyo, Japan) and cellSens Dimension imaging software (Olympus, Hamburg, Germany).

A follow-up was conducted by surveying the veterinarians or owners. The survey included assessment of the incidence of local recurrence and the survival time up to 2 years after the diagnosis.

The results were analysed statistically. A Spearman rank correlation analysis was used to detect the correlations between the evaluated morphological and immunohistochemical features (r: Spearman rank correlation coefficient) of CCH. The number of histiocytic sarcomas was too low to perform a reliable statistical analysis. The correlations were considered statistically significant when P < 0.05. The statistical analysis was performed using Statistica software (StatSoft 10 eng. 32 bit, Tulsa, OK).

## Results

**Fig. 1:**
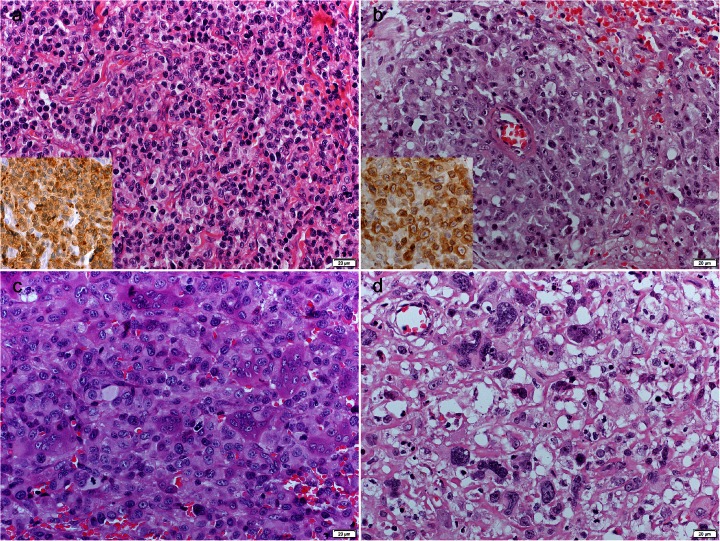
(**a**) Epitheliotropic T-cell lymphoma, dog. The tumour was morphologically diagnosed as CCH, reclassified after immunohistochemistry. The tumour cells expressed CD3 (inset). HE. Inset: immunoperoxidase stain; DAB substrate; Mayer’s haematoxylin counterstain. (**b**) Non-epitheliotropic T-cell lymphoma, dog. The tumour was morphologically diagnosed as canine cutaneous histiocytic sarcoma, reclassified after immunohistochemistry. The tumour cells expressed CD3 (*inset*). HE. Inset: immunoperoxidase stain; DAB substrate; Mayer’s haematoxylin counterstain. (**c**) Unclassified sarcoma, dog. The tumour was morphologically classified as canine cutaneous histiocytic sarcoma and was negative for MHCII, CD18, CD3 and CD79. HE. (**d**) Unclassified sarcoma, dog. The tumour was morphologically classified as canine cutaneous histiocytic sarcoma and was negative for MHCII, CD18, CD3 and CD79. HE

The histiocytic origin of the tumour cells (MHCII+, CD18+, CD79αcy-, CD3-) was confirmed in 65 CCHs and 4 cutaneous histiocytic sarcomas. Two tumours diagnosed histologically as CCH showed expression of CD3; therefore, their final diagnosis was epitheliotropic T-cell lymphoma (Fig. 1a). Two other tumours diagnosed histologically as CCH showed expression of CD79αcy; therefore, their final diagnosis was plasmacytoma. In 2 of the cases of CCH, the origin of the tumour cells remained undetermined, as they showed expression of MHCII but were negative for CD18, CD79αcy and CD3. One tumour diagnosed histologically as a histiocytic sarcoma showed expression of CD79αcy; therefore, its final diagnosis was plasmacytoma. One other tumour diagnosed histologically as a histiocytic sarcoma showed expression of CD3; therefore, the final diagnosis was non-epitheliotropic T-cell lymphoma (Fig. 1b). Eight tumours diagnosed histologically as histiocytic sarcomas remained undetermined after immunohistochemistry (Fig. 1c and d). For all of the evaluated tumours, the detailed immunophenotypes of the tumour cells are summarised in Table 2.

**Table 2:** Histologic diagnosis and labelling panel results (final diagnosis) for evaluated canine cutaneous tumours

Histologic diagnosis	No. Tumours	MHCII	CD18	CD79αcy	CD3	E-cadherin	Final diagnosis
CCH	65	+	+	-	-	+	CCH
CCH	1	-	-	+	-	-	plasmacytoma
CCH	1	+	+	+	-	+	plasmacytoma
CCH	1	+	+	-	+	-	epitheliotropic T-cell lymphoma
CCH	1	-	-	-	+	+	epitheliotropic T-cell lymphoma
CCH	1	+	-	-	-	ND	undetermined
CCH	1	+	-	-	-	-	undetermined
histiocytic sarcoma	2	+	+	-	-	+	HS
histiocytic sarcoma	2	+	+	-	-	-	HS
histiocytic sarcoma	1	+	+	+	-	+	plasmacytoma
histiocytic sarcoma	6	-	-	-	-	ND	undetermined
histiocytic sarcoma	2	+	-	-	-	ND	undetermined
histiocytic sarcoma	1	+	-	-	+	ND	non-epitheliotropic T-cell lymphoma

### CCH

**Fig. 2:**
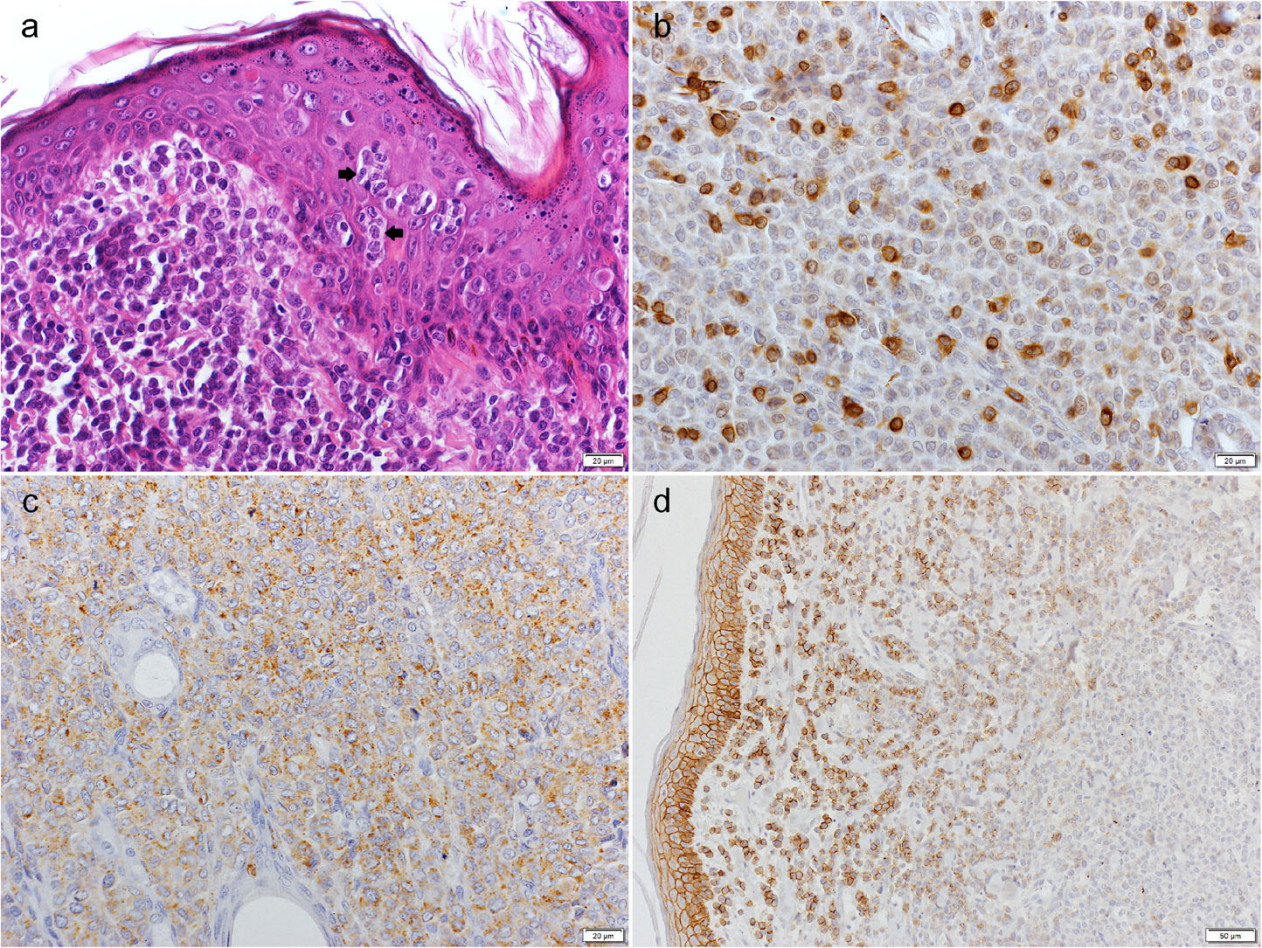
CCH, dog. (**a**) Intra-epidermal nests of neoplastic cells (*arrows*). HE. (**b**) Neoplastic histiocytoma cells accompanied by diffuse T-lymphocytes (CD3+). Immunoperoxidase stain; DAB substrate; Mayer’s haematoxylin counterstain. (**c**) Neoplastic histiocytoma cells showing diffuse cytoplasmic MHCII expression. Immunoperoxidase stain; DAB substrate; Mayer’s haematoxylin counterstain. (**d**) Expression of E-cadherin in tumour cells, with strong expression in the subepidermal area of the tumour that gradually decreased in the deeper parts of the tumour. Immunoperoxidase stain; DAB substrate; Mayer’s haematoxylin counterstain

The tumours occurred exclusively as solitary cutaneous nodules. The localisation of the tumours and the characteristics of the affected dogs are summarised in Table 3. The tumours were poorly circumscribed and nonencapsulated; localised under the epidermis; and extending to the deep dermis or, not uncommonly, to the subcutis. The neoplastic cells formed a solid mass with occasional sheet formation. The cellular arrangement was superficially loose and dense in the deeper areas of the tumour. The neoplastic cells were round, oval or polygonal, with abundant and slightly eosinophilic cytoplasm and indistinct cell borders. The nuclei were round or reniform and occasionally indented or folded. The chromatin was finely dispersed (superficial areas of the tumour) or marginated (deep areas of the tumour), with single nucleoli. The mitotic index was variable; most CCHs showed 1–4 mitotic figures per HPF (magnification 400x), but single tumours showed up to 9 mitotic figures per HPF. The mitotic index was negatively correlated with the intensity of the lymphocytic infiltration (*r* = −0.259; *P* = 0.037). Prominent necrotic foci were found in 38 % of the cases, but necrosis or apoptosis of single tumour cells was common and associated with the lymphocytic infiltration. The epidermal changes included ulceration (92 % of the cases), parakeratosis (21 % of the cases), intra-epidermal aggregates of tumour cells (19 % of the cases, Fig. 2a) and hydropic degeneration (15 % of the cases). Numerous capillary vessels (angiogenesis) within the tumour (primarily in the subepidermal areas) were evident in 37 % of the cases. Reticulin fibres surrounded the tumour cells in variable amounts.

**Table 3:** Clinical characteristics of the evaluated cases of CCH

Sex	Male	50 % of cases
	Female	50 % of cases
Localisation	Head, neck	47 % of cases
	Trunk	11 % of cases
	Limbs	42 % of cases
Age	Under 3 years old	69 % of cases
	Over 3 years old	31 % of cases
Breed (>4 cases)	Boxer, Yorkshire Terrier, mongrel	
Breed (1–4 cases)	Labrador Retriever, American Staffordshire Terrier, Dachshund, German Shepherd, French Bulldog, Beagle, Border Collie, Scenthound, Schnauzer, Russian Terrier, West Highland White Terrier, Shar-Pei, Cocker Spaniel, Fox Terrier, Bordeaux Mastiff, Pinscher, Poodle, Bull Terrier, Golden Retriever, Great Dane

The T-lymphocyte infiltrate was predominantly focal and diffuse (77 % of the cases, Fig. 2b). B-lymphocytes and plasma cells predominantly formed large foci (46 % of the cases) or occurred as single cells (35 % of the cases). The lymphocytic infiltration was minimal in 13 CCHs (20 %), moderate in 27 CCHs (42 %), marked in 17 CCHs (26 %) and massive in 8 CCHs (12 %). The T- and B-lymphocytes (with plasma cells) were positively correlated (*r* = 0.342; *P* = 0.005). Neutrophils were mostly observed as small or large foci and were commonly associated with epidermal ulceration and necrotic foci within the tumour. Eosinophils were noted in 21 % of the cases, as either single cells or small foci. Mast cells were confirmed in 28 % of the cases, mostly as small foci, and were localised within the tumour or at the tumour periphery. The mast cells within the tumour and at the tumour periphery were positively correlated (*r* = 0.697; *P* = 0.000). The number of mast cells within the tumour was negatively correlated with the intensity of the lymphocytic infiltration (*r* = −0.277; *P* = 0.026).

A quantitative analysis of MHCII, CD18 and E-cadherin expression is summarised in Table 4. Two primary MHCII expression patterns were observed in the tumour cells: diffuse cytoplasmic, with distinct juxtanuclear foci only occasionally observed, and membranous (rim-like). In approximately half of the tumours, both expression patterns were observed, but with different proportions of cells showing either a cytoplasmic or a membranous labelling pattern. MHCII expression was cytoplasmic in 32 % of the CCHs (Fig. 2c), predominantly cytoplasmic in 31 % of the CCHs, predominantly membranous in 20 % of the CCHs and membranous in 17 % of the CCHs. The MHCII expression pattern was positively correlated with the intensity of the lymphocytic infiltration (*r* = 0.574; *P* = 0.000) and negatively correlated with the number of mast cells within the tumour (*r* =−0.453; *P* = 0.000) and at the tumour periphery (*r* =−0.293; *P* = 0.018). The CD18 expression pattern was either strictly membranous or membranous with slight cytoplasmic accentuation. Strong variations between the labelling intensities were noted. Strong expression was confirmed in 11 % of the CCHs; moderate, in 67 % of the CCHs; and weak, in 22 % of the CCHs. The number of tumour cells expressing CD18 was positively correlated with the intensity of the CD18 expression (*r* = 0.642; *P* = 0.000), the intensity of the lymphocytic infiltration (*r* = 0.303; *P* = 0.014), the number of tumour cells expressing MHCII (*r* = 0.638; *P* = 0.000) and the MHCII expression pattern (*r* = 0.284; *P* = 0.022). The E-cadherin expression pattern was membranous. The positive cells were usually subepidermally situated, gradually losing E-cadherin expression in the deeper parts of the tumour (Fig. 2d). There were single cases in which the E-cadherin expression was limited to a few scattered cells within the tumour. The E-cadherin expression was negatively correlated with the intensity of the lymphocytic infiltration (*r* =−0.263; *P* = 0.036).

**Table 4:** Quantitative analysis of MHCII, CD18 and E-cadherin expression in CCH

Percentage of tumour cells with immunolabelling		Less than 20%	20-50%	51-80%	More than 80 %
Number (percentage) of histiocytoma cases	MHCII	1 (2 %)	2 (3 %)	8 (12 %)	54 (83 %)
	CD18	6 (9 %)	3 (5 %)	17 (26 %)	39 (60 %)
	E-cadherin	18 (28 %)	23 (35 %)	23 (35 %)	1 (2 %)

Follow-up was available in 44 of the CCH cases. Recurrence was noted in two cases, but a subsequent histopathological examination was not performed. Two dogs (a 10-year-old Boxer and a 10-year-old American Staffordshire Terrier) died within one year after the surgery, but the reason for death was not associated with CCH. Necropsies were not performed.

### Histiocytic sarcoma

**Fig. 3:**
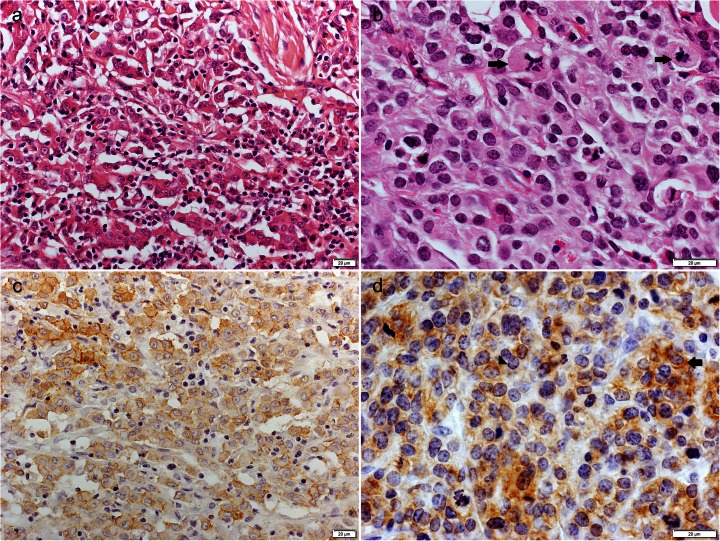
Canine cutaneous histiocytic sarcoma, dog. (**a**) Polygonal to stellate neoplastic cells with short cytoplasmic projections and a tendency to coalesce. HE. (**b**) Round to polygonal neoplastic cells with variable amounts of slightly eosinophilic cytoplasm and frequent mitotic figures, often bizarre (*arrows*). HE. (**c**) Tumour cells showing cytoplasmic E-cadherin expression. Immunoperoxidase stain; DAB substrate; Mayer’s haematoxylin counterstain. (**d**) Tumour cells showing cytoplasmic (*arrowhead*) and membranous (*arrow)* expression of E-cadherin. Immunoperoxidase stain; DAB substrate; Mayer’s haematoxylin counterstain

The first case of histiocytic sarcoma occurred as a solitary cutaneous nodule situated in the area of the elbow in an 11-month-old mongrel male. The tumour was poorly circumscribed, nonencapsulated, localised under the epidermis, and extending to the deep dermis. The neoplastic cells formed a solid mass, obliterating the adnexa, with a dense cellular arrangement and abundant reticulin fibres. The neoplastic cells were round to polygonal and oval to fusiform, with abundant, slightly eosinophilic cytoplasm and indistinct cell borders. The nuclei were oval, round or reniform, with marginated chromatin and indistinct nucleoli. The mitotic index was 16. The second case of histiocytic sarcoma occurred as a solitary cutaneous nodule situated in the thigh area in an 8-year-old Cane Corso male. The tumour was poorly circumscribed and nonencapsulated, localised in the dermis, and extending deeply into the subcutis and focally to the epidermis. The neoplastic cells formed sheets and cords, with a loose cellular arrangement and a moderate amount of reticulin fibres. The neoplastic cells were polygonal and stellate, with short cytoplasmic projections. The neoplastic cells had a tendency to coalesce (Fig. 3a). The abundant cytoplasm was deeply eosinophilic. The nuclei were round to polygonal, with marginated chromatin and large, distinct nucleoli. The mitotic index was 3. The tumour was focally necrotic. The third case of histiocytic sarcoma occurred as multiple cutaneous nodules in a 6-year-old Shar-Pei female. The tumour was poorly circumscribed and nonencapsulated, localised under the epidermis and extending deeply into the subcutis. The neoplastic cells formed a diffuse mass with a dense or occasionally loose cellular arrangement and a moderate amount of reticulin fibres. The neoplastic cells were round to polygonal, with variable amounts of slightly eosinophilic cytoplasm (Fig. 3b). The neoplastic cells had a tendency to coalesce, and multinucleated cells were noted occasionally. The nuclei were round to polygonal, with coarse or densely packed chromatin and indistinct single nucleoli. The mitotic index was 12. The fourth case of histiocytic sarcoma occurred as a solitary cutaneous nodule located in the abdominal area in a 7-year-old Pointer female. The tumour was poorly circumscribed and nonencapsulated and was localised in the subcutis. The neoplastic cells formed a solid mass with a dense cellular arrangement and abundant reticulin fibres. The neoplastic cells were polygonal, with abundant, deeply eosinophilic cytoplasm. The nuclei were elongated, with marginated chromatin and 1–2 nucleoli of variable sizes. The mitotic index was 2. The tumour was focally necrotic.

In all cases, the tumour cells were accompanied by numerous T-lymphocytes (focal and diffuse infiltrate) and less numerous B-lymphocytes (focal infiltrate or single cells). Neutrophils formed large foci, mostly related to the necrotic areas. Mast cells and eosinophils were observed sporadically. The MHCII expression pattern was membranous or cytoplasmic and confirmed in more than 80 % of the tumour cells (three cases) or in 51-80 % of the tumour cells (one case). The CD18 expression pattern was membranous and confirmed in more than 80 % of the tumour cells (two cases), in 51-80 % of the tumour cells (one case) or in 20-50 % of the tumour cells (one case). The tumour cells were E-cadherin positive in two cases. The expression of E-cadherin was membranous (Fig. 3c; confirmed in more than 80 % of the tumour cells) or membranous and cytoplasmic (Fig. 3d; confirmed in 51–80 % of the tumour cells).

In the first case of histiocytic sarcoma, the tumour recurred, and the dog died within a year of the diagnosis. According to the survey, the cause of death was related to the tumour, but neither a histopathological examination of the recurrent tumour nor necropsy was performed. In the second case, the tumour recurred within a year of the diagnosis, and in the following months, multiple nodules developed in the neighbouring areas. One of the tumours was excised surgically, and a histopathological examination confirmed the histiocytic sarcoma. The dog was still alive at the time of the survey (2 years after the initial diagnosis). In the third case, the dog was euthanised due to a poor prognosis following the diagnosis. In the fourth case, the dog died within two years of the diagnosis. According to the survey, the cause of death was related to the tumour, but a necropsy was not performed.

## Discussion

The present study revealed that a routine histopathological examination based solely on morphology is insufficient for diagnosing CCH. Six of the 71 tumours (8 %) that were diagnosed as CCH had to be reclassified after an immunohistochemical examination. The tumours misdiagnosed as CCH included epitheliotropic lymphoma and plasmacytoma. Lee Gross et al. revealed that CCH and cutaneous T-cell lymphoma can mimic each other perfectly; thus, immunohistochemistry is often required for differentiation (2005). Plasmacytoma (or B-cell lymphoma) has also previously been misdiagnosed as CCH (Fernandez et al. 2005). The other differential diagnoses for CCHs include Merkel cell tumours, amelanotic melanomas, poorly granulated mast cell tumours and transmissible venereal tumours (Lee Gross et al. 2005). Two of the 71 tumours (3 %) showed expression of MHCII and were negative for the other evaluated antigens, therefore remaining undetermined after the immunohistochemical examination. A possible diagnosis for these tumours is plasmacytoma, as approximately 20 % of plasmacytomas lack CD79 expression (Moore et al. 2000).

The majority of the evaluated CCHs occurred in dogs under 3 years of age and were situated in the areas of the head, neck or limbs, which is consistent with previous reports (Schwens et al. 2011; Moore 2014). The most affected breeds included Boxers, Yorkshire Terriers and mongrels, but this most likely reflects the breeds’ prevalence in society, rather than a true breed predilection. However, the Boxer is regarded (together with Dachshunds) as a predisposed breed (Moore 2014). The morphology of CCH described in the present study is consistent with previous reports (Taylor et al. 1969; Moore et al. 1996; Lee Gross et al. 2005; Schwens et al. 2011; Moore 2014). However, the intra-epidermal aggregates of the tumour cells were confirmed in only a small percentage of the cases in the current study, whereas in previous studies, neoplastic intra-epidermal aggregates were considered to be a frequent feature of CCH (Moore et al. 1996; Lee Gross et al. 2005). In our opinion, evaluation of the epidermis covering a CCH could be problematic due to very frequent massive ulceration. However, intra-epidermal invasion by tumour cells is most likely not as prevalent as previously reported, which has also been suggested by another author (Moore 2014). The other epidermal changes described in the present study, such as parakeratosis and hydropic degeneration, are commonly associated with a response to chronic stimuli (McGavin and Zachary 2012). In several of the cases of CCH in the present study, the epidermal ulceration was accompanied by distinct angiogenesis in the adjacent tumour mass. Such angiogenesis is associated with inflammation and is stimulated by macrophages and dendritic cells (Dong et al. 2009). The mitotic index of the evaluated CCHs was highly variable but typically moderate. There were also tumours with surprisingly high mitotic indexes. Previous reports have described the mitotic activity in CCH as moderate (Lee Gross et al. 2005) or as highly variable but often high (Moore 2014). CCH shows the highest mitotic index compared with other benign cutaneous tumours in dogs (Martin de Las Mulas et al. 1999). In the present study, the mitotic index decreased with an increase in the intensity of the lymphocytic infiltration. We suggest that the infiltrating lymphocytes may contribute to the inhibition of proliferation in CCH. The decrease in the mitotic index with tumour regression was previously shown in CCH (Baines et al. 2000) and in transmissible venereal tumours (Mukaratirwa et al. 2006). However, no significant differences in Ki67 expression at the different stages of CCH regression have been shown (Pires et al. 2013a).

T-lymphocytes frequently accompany CCH tumour cells, taking part in the immune-mediated spontaneous regression (Cockerell and Slauson 1979). In the present study, T-lymphocytes were numerous in most of the CCHs and definitively outnumbered B-lymphocytes. As previously reported, most of the lymphocytes infiltrating CCHs were CD3+ and CD8+, whereas B-lymphocytes and CD4 lymphocytes were encountered less frequently (Moore et al. 1996). The results of the present study showed that the T- and B-lymphocytes were positively correlated, which suggests a synergistic role for the two lymphocytic populations in tumour regression. B-lymphocytes are able to mediate tumour regression, which has been shown in adoptive cancer immunotherapy (Li et al. 2009). Kipar et al. reported that the numbers of B-lymphocytes and plasma cells increased slightly within focal inflammatory infiltrates in CCH (1998). Pires et al. noted an increase in both T- and B-lymphocytes in regressing CCHs (2013b). In the present study, neutrophils were primarily associated with the necrotic foci, but neither the number of neutrophils nor the incidence of the necrotic foci was related to the intensity of the lymphocytic infiltration. The mechanism of tumour regression is connected with CD8+ lymphocytes, which cause the apoptosis or necrosis of tumour cells (Barry and Bleackley 2002). However, a previous study reported that the apoptotic rate was not increased in regressing histiocytomas, most likely due to the rapid turnover of the apoptotic cells in the tissue (Kaim et al. 2006). In the present study, eosinophils were found only incidentally, which is consistent with previous reports (Lee Gross et al. 2005). Furthermore, mast cells were noted in only several of the CCHs, and the number of mast cells decreased with the intensity of the lymphocytic infiltration. In transmissible venereal tumours, there are higher mast cell counts within regressing tumours than within progressing tumours (Mukaratirwa et al. 2006). T cell-derived mediators, such as the beta-chemokines, directly induce mast cell degranulation (Mekori and Metcalfe 1999). Degranulated mast cells are not recognisable with toluidine blue, which may explain why these cells were found to be prevalent in the CCHs with minimal or moderate lymphocytic infiltration. To evaluate the true incidence of mast cells in CCH, specific markers should be used, such as mast cell tryptase.

The great majority of evaluated CCHs showed expression of MHCII in nearly all of the tumour cells. MHCII, together with CD18, is considered to be a reliable marker for the leukocytic origin of tumour cells (Fernandez et al. 2005). However, 17 % of the cases showed MHCII expression in only some of the tumour cells, which has also been noted by other authors (Kipar et al. 1998). The MHCII labelling pattern in CCH tumour cells was previously detected as focal juxtanuclear cytoplasmic labelling and/or rim-like labelling along the cell periphery (Kipar et al. 1998; Pires et al. 2013b). In the present study, mixed labelling patterns were additionally observed, as approximately half of the evaluated tumours consisted of a mixed tumour cell population showing an either cytoplasmic or membranous labelling pattern. The transport of the MHCII molecules from the cytoplasmic vesicles to the cell membrane reflects the maturation process of dendritic cells after antigenic stimulation (Van Niel et al. 2008) and appears to be crucial for the onset of CCH regression (Kipar et al. 1998). In the present study, the MHCII expression pattern was positively correlated with the intensity of the lymphocytic infiltration, which supports previous observations (Kipar et al. 1998; Pires et al. Pires et al. 2013b). The presentation of a yet-unknown antigen together with MHCII might induce the priming and activation of T-cells and the development of tumour-specific cytotoxic T-cells (Kipar et al. 1998), suggesting that CCH is a self-limiting neoplastic disease in which tumour cells cause immune-mediated self-destruction.

The present study demonstrated that the number of tumour cells showing CD18 expression was positively correlated with the intensity of the expression and that a significant number of CCHs showed CD18 expression in only a small number of the tumour cells. A reasonable explanation for this level of expression may be a loss of antigenicity due to prolonged fixation (Haines and Chelack 1991). Meanwhile, the positive correlation between MHCII and CD18 expression in tumour cells suggests that less differentiated tumour cells can be negative for the markers characteristic of their cell line. The results of this study showed that the number of tumour cells expressing CD18 was positively correlated with the intensity of the lymphocytic infiltration, indicating that well-differentiated tumour cells are more prone to regression.

E-cadherin is expressed by both normal Langerhans cells and CCH tumour cells (Jakob et al. 1999; Baines et al. 2008). Although E-cadherin is regarded as a constant feature of the CCH immunophenotype, CCHs lacking E-cadherin expression have also been observed (Moore 2014). In the present study, most tumours showed E-cadherin expression in some of the tumour cells, and in single cases, there were only few E-cadherin-positive cells. In these cases, the E-cadherin expression could be easily overlooked. Therefore, the expression of E-cadherin is of limited diagnostic value in CCH. According to Valli et al., Langerhans cells are the only leukocytes that express E-cadherin (2002). However, in the present study, one of the misdiagnosed plasmacytomas and one epitheliotropic lymphoma showed E-cadherin expression. Therefore, E-cadherin expression is not specific to Langerhans cells and cannot distinguish canine cutaneous round cell tumours, which was also confirmed in a previous study (Ramos-Vara and Miller 2011). The present study revealed that E-cadherin expression decreased with an increase in the intensity of the lymphocytic infiltration. The decrease in E-cadherin expression in normal Langerhans cells is connected with the cells’ migration to the lymph nodes and reflects their maturation after antigenic stimulation (Wang et al. 1999; Baines et al. 2008). Therefore, the CCH tumour cells most likely undergo a maturation process in a similar manner as normal Langerhans cells do, which is also supported by other authors (Baines et al. 2000; Pires et al. 2009). The loss of E-cadherin expression in CCH suggests that the tumour cells of regressing tumours can migrate to the draining lymph nodes. Little is known about the migration of CCH tumour cells to the draining lymph nodes, and the true incidence is most likely underestimated (Moore et al. 1996).

CCH is a benign tumour, and the recurrence rate is extremely low (Moore 2014). In the present study, 2 of the 44 CCHs with available follow-up information (5 %) recurred after surgical excision, so although the recurrence rate is low, it should not be regarded as incidental. However, the lack of histopathological examinations of the recurrent tumours limits the interpretation of this finding.

The present study revealed that immunohistochemistry is absolutely required in diagnosing canine cutaneous histiocytic sarcomas. Ten of the 14 tumours (71 %) diagnosed as histiocytic sarcomas had to be reclassified after the immunohistochemical examination, and most of them remained undetermined, as they were negative for all or nearly all of the evaluated markers. The reason for the high rate of misdiagnoses may be either the poorly specified diagnostic criteria or strong morphological variations in this group of cutaneous tumours. Canine histiocytic sarcomas can mimic other spindle cell or round cell sarcomas; therefore, immunophenotypic evaluation is required (Affolter and Moore 2002). In the current study, the tumours misdiagnosed as histiocytic sarcomas included, other than the undetermined tumours, non-epitheliotropic lymphoma and plasmacytoma. In contrast to non-epitheliotropic lymphoma, plasmacytoma has been included in the differential diagnoses of round-cell-predominant histiocytic sarcomas by certain authors (Lee Gross et al. 2005). Undetermined tumours should be subjected to further immunohistochemistry, such as analysis of smooth-muscle actin, desmin, vimentin, von Willebrand factor or neuron-specific enolase, before a final diagnosis of “poorly differentiated sarcoma” should be made.

The small number of cutaneous histiocytic sarcomas that were evaluated does not allow any generalisations or conclusions to be made about their incidence, their breed or sex prevalence, or their preferred locations. However, the difficulty in collecting more tumour samples indicates that these tumours are rare, especially compared with their benign counterpart, or are frequently misdiagnosed and therefore underestimated. Furthermore, the histiocytic sarcomas described in this report showed marked morphological diversity. The cellular shape varied from round or polygonal to fusiform. None of the described tumours was characterised by the presence of all three cellular populations of round, spindle and multinucleated giant cells, as described previously (Affolter and Moore 2002). Regarding the shape of the predominating cells, a spindle-cell-predominant and round-cell-predominant pattern of histiocytic sarcoma was previously distinguished (Lee Gross et al. 2005). In Flat-Coated Retrievers, the histiocytic subtype and histiocytic-spindle-pleomorphic subtype have been described (Constantino-Casas et al. 2011). The evaluated histiocytic sarcomas showed pleocellular or predominantly lymphocytic inflammatory infiltrates. Histiocytic sarcomas tend to have more cellular variability in the accompanying inflammatory cells compared with other soft tissue sarcomas (Valli et al. 2002). In the present study, the histiocytic sarcomas showed MHCII and CD18 expression, as previously described (Affolter and Moore 2002; Constantino-Casas et al. 2011). However, two of the evaluated histiocytic sarcomas were also E-cadherin positive. E-cadherin is a lineage-associated marker for Langerhans cells (Moore 2014). The cutaneous histiocytic sarcoma is derived from interstitial dendritic cells and therefore lacks expression of E-cadherin (Lee Gross et al. 2005). Ramos-Vara and Miller described E-cadherin expression in five cutaneous histiocytic sarcomas, but without immunohistochemical confirmation of the histiocytic origin of the tumour cells (2011). The results of the present study suggest that cutaneous histiocytic sarcomas may possibly be derived not only from interstitial dendritic cells but also from Langerhans cells.

In conclusion, a routine histopathological examination is insufficient for diagnosing CCHs and canine cutaneous histiocytic sarcomas. Immunohistochemistry, including analysis of MHCII, CD18 and the lymphocytic markers CD3 and CD79, should be performed in questionable cases of CCH and in any case of canine cutaneous histiocytic sarcoma. E-cadherin expression is of limited diagnostic value in CCH. Certain canine cutaneous histiocytic sarcomas are E-cadherin positive and are therefore possibly derived from Langerhans cells. The spontaneous regression of CCH, determined by T- and B-lymphocyte infiltration, is connected with a decrease in the mitotic index, an increase in the transport of MHCII molecules from the cytoplasm to the cell membrane, and a loss of E-cadherin expression in the tumour cells. The present study highlights the practical aspects of diagnosing canine cutaneous histiocytic tumours.
